# Morphological Variations of the Anterior Cerebral Artery: A Systematic Review with Meta-Analysis of 85,316 Patients

**DOI:** 10.3390/diagnostics15151893

**Published:** 2025-07-28

**Authors:** George Triantafyllou, Ioannis Paschopoulos, Katerina Kamoutsis, Panagiotis Papadopoulos-Manolarakis, Juan Jose Valenzuela-Fuenzalida, Juan Sanchis-Gimeno, Alejandro Bruna-Mejias, Andres Riveros-Valdés, Nikolaos-Achilleas Arkoudis, Alexandros Samolis, George Tsakotos, Maria Piagkou

**Affiliations:** 1 Department of Anatomy, School of Medicine, Faculty of Health Sciences, National and Kapodistrian University of Athens, 11527 Athens, Greece; georgerose406@gmail.com (G.T.); johnpascho@gmail.com (I.P.); katerina.kamoutsis@gmail.com (K.K.); p.papado@gmail.com (P.P.-M.); alexsamolis@me.com (A.S.); gtsakotos@gmail.com (G.T.); 2 “VARIANTIS” Research Laboratory, Department of Clinical Anatomy, Masovian Academy in Płock, 09400 Płock, Poland; 3 Department of Neurosurgery, General Hospital of Nikaia-Piraeus, 18454 Athens, Greece; 4 Departamento de Morfología, Facultad de Medicina, Universidad Andrés Bello, Santiago 8370146, Chile; juan.kine.2015@gmail.com; 5 Giaval Research Group, Department of Anatomy and Human Embryology, Faculty of Medicine, University of Valencia, 46001 Valencia, Spain; juan.sanchis@uv.es; 6 Department of Sciences and Geography, Faculty of Natural and Exact Sciences, Universidad de Playa Ancha, Valparaíso 2360072, Chile; alejandro.bruna@upla.cl; 7 Department of Morphological Sciences, Faculty of Sciences, Universidad San Sebastián, Lientur 1457, Concepción 4080871, Chile; andres.riveros@uss.cl; 8 Research Unit of Radiology and Medical Imaging, National and Kapodistrian University of Athens, 12462 Athens, Greece; nick.arkoudis@gmail.com; 9 Second Department of Radiology, General University Hospital Attikon, National and Kapodistrian University of Athens, 12462 Athens, Greece

**Keywords:** anterior cerebral artery, variation, neuroradiology, evidence-based anatomy, meta-analysis

## Abstract

**Background**: The anterior cerebral artery (ACA), a critical component of the cerebral arterial circle, exhibits substantial morphological variability. While previous studies have explored ACA morphology using cadaveric and imaging methods, a comprehensive meta-analysis incorporating the latest evidence is lacking. **Methods**: Following current guidelines, a systematic review and meta-analysis were performed across four major databases, supplemented by the gray literature and targeted journal searches. Ninety-nine studies, encompassing 85,316 patients, met the inclusion criteria. Statistical analyses were conducted using R, applying random effects models to estimate pooled prevalence and morphometric parameters. **Results**: The pooled prevalence of typical ACA morphology was 93.75%, whereas variants were noted in 6.25% of cases. The predominant variation identified was the accessory ACA (aACA) (1.99%), followed by unilateral absence of the A1 segment (1.78%), with the latter being more frequently recognized in imaging studies (*p* < 0.0001). Rare variants encompassed azygos ACA (azACA) (0.22%), fenestrated ACA (fACA) (0.02%), and bihemispheric ACA (bACA) (0.02%). The mean diameter and length of the A1 segment were measured at 2.10 mm and 14.24 mm, respectively. Hypoplasia of the A1 segment (<1 mm diameter) was recorded in 3.15% of cases. The influences of imaging modality, laterality, and population distribution on prevalence estimates were minimal. No significant publication bias was detected. **Conclusions**: Although infrequent, variants of the ACA possess significant clinical importance attributable to their correlation with aneurysm formation and the impairment of collateral circulation. The aACA and the absence of the A1 segment emerged as the most common variations. This meta-analysis presents an updated and high-quality synthesis of ACA morphology, serving as a valuable reference for clinicians and anatomists.

## 1. Introduction

The variability of the cerebral arterial circle is often described through (cadaveric) dissection and imaging techniques. Documenting the typical anatomy of the brain’s vascular supply and potential morphological variants is exceptionally straightforward with computed tomography (CTA), magnetic resonance (MRA), or digital subtraction angiography (DSA) [1,2,3].

The anterior circulation of the brain originates from the internal carotid artery (ICA) system, comprising the anterior and middle cerebral arteries (ACA and MCA). According to Gray’s Anatomy and Bergman’s Comprehensive Encyclopedia of Human Anatomic Variations, the ACA is divided into three parts: from its origin to the junction with the anterior communicating artery (AComA)—the A1 segment; from the intersection with the AComA to the origin of the callosomarginal artery (CMA)—the A2 segment; and distal to the origin of the CMA—the A3 segment [4,5]. The ACA’s course is also significant, as it initially passes anteromedially to the optic nerve (ON), and then travels in the great longitudinal fissure and around the genu of the corpus callosum [4].

Several ACA variations have been described, mainly for the A1 and proximal A2 segments. These variants include A1 hypoplasia or absence, A1 fenestration, accessory A2 (triplicated ACA or median artery of corpus callosum), azygos ACA (azACA), and bihemispheric ACA (bACA) [5]. All variations in the ACA were previously associated with aneurysm formation [6]. However, it is essential to mention that variations are more frequently located at the AComA complex [7].

Although research on ACA morphology has expanded recently, only Fotakopoulos et al. [8] have published a systematic review with a meta-analysis. In contrast, our study identified a substantially larger dataset and a broader spectrum of ACA variants. This meta-analysis aims to provide a comprehensive, evidence-based overview of ACA variability using current anatomical and statistical standards.

## 2. Materials and Methods

The systematic review with a meta-analysis adhered to the guidelines set forth by the Evidence-based Anatomy Workgroup for anatomical meta-analysis [9] and the PRISMA 2020 for systematic reviews (see Supplementary Materials) [10], similar to previous studies [11,12]. The study’s protocol was not registered in any online database. The figures were obtained from the General Hospital of Nikaia-Piraeus following ethical approval (approval number: 56485; date: 13 November 2024).

The literature search was performed using the online databases PubMed, Google Scholar, Scopus, and Web of Science until April 2025. The following terms were used in various combinations: *“anterior cerebral artery,” “anterior communicating artery,” “variation,” “anterior circulation,” “cadaveric study,” “imaging study,” and “radiological study.”* Furthermore, the references of all included articles were reviewed, the gray literature was investigated, and a comprehensive search of key anatomical journals (Annals of Anatomy, Clinical Anatomy, Journal of Anatomy, Anatomical Record, Surgical and Radiological Anatomy, Folia Morphologica, European Journal of Anatomy, Morphologie, Anatomical Science International, and Anatomy and Cell Biology) was conducted. The inclusion criteria consisted of studies that reported the prevalence of ACA variants. Case reports, conference abstracts, animal studies, and studies presenting irrelevant or insufficient data were excluded.

Three independent reviewers (GTr, IP, and KK) searched the literature and extracted data into Microsoft Excel sheets. The results were compared, and the other authors resolved any discrepancies. The Anatomical Quality Assurance (AQUA) tool, developed by the Evidence-based Anatomy Workgroup for anatomical reviews [13], was utilized to assess the risk of bias for each article.

A statistical meta-analysis was conducted using the open-source R programming language and RStudio software (version 4.3.2), employing the “meta” and “metafor” packages by a single researcher (GTr). The pooled prevalence was calculated utilizing inverse variance and random effects models. The proportion (prevalence) meta-analysis was performed using the Freeman–Tukey double arcsine transformation, the DerSimonian–Laird estimator for the between-study variance tau^2^, and the Jackson method for the confidence interval of tau^2^ and tau. The mean (mean diameter) meta-analysis was executed using the untransformed (raw) means, the restricted maximum likelihood estimator for tau^2^, and the Q-Profile method for confidence intervals of tau^2^ and tau. Furthermore, several subgroup analyses were conducted to identify variables (*geographic distribution, laterality, or imaging technique*) influencing the estimated pooled prevalence and mean. A *p*-value of less than 0.05 was considered statistically significant. Cochran’s Q statistic was employed to evaluate the presence of heterogeneity across studies, while the Higgins I^2^ statistic quantified this heterogeneity. A Cochran’s Q *p*-value < 0.10 was regarded as significant. Higgins I^2^ values between 0 and 40% were classified as negligible, 30–60% as moderate heterogeneity, 50–90% as substantial heterogeneity, and 75–100% as considerable heterogeneity. To assess the presence of a small-study effect (the phenomenon that smaller studies may exhibit differing effects compared to larger studies), the DOI plot with the LFK index was generated for the proportions meta-analysis [14], and the Funnel Plot with the Thomson–Sharp test was utilized for the means meta-analysis [15].

## 3. Results

### 3.1. Search Analysis

**Figure 1:**
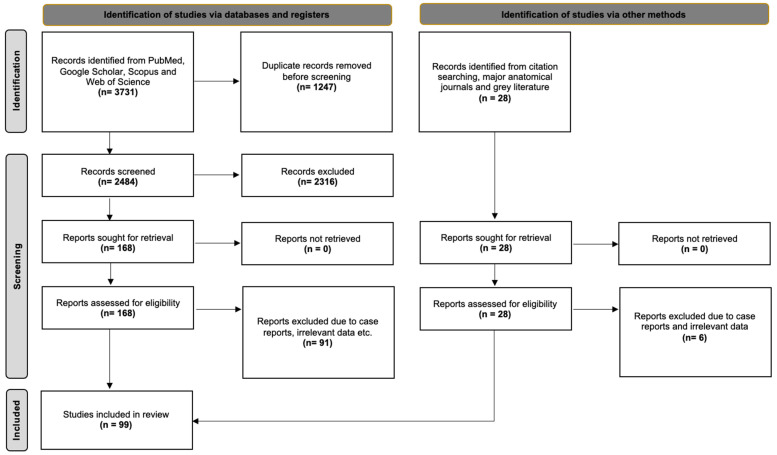
The search analysis flow chart according to the PRISMA 2020 guidelines.

The database search yielded 3731 articles exported to Mendeley version 2.10.9 (Elsevier, London, UK). After excluding duplicate and irrelevant papers through title and abstract screening, 168 studies were subjected to full-text retrieval and examination. Ultimately, 77 studies were deemed eligible for systematic review. Additionally, 22 studies were identified through our secondary investigation, which included references, the gray literature, and an extensive search of anatomical journals. Therefore, 99 studies were included in our systematic review with meta-analysis. Figure 1 summarizes the flow diagram of our search analysis according to the PRISMA 2020 guidelines.

### 3.2. Characteristics of Eligible Studies

A total of ninety-nine (99) studies were included in this analysis, encompassing a combined cohort of 85,316 patients. The average sample size per article was 862 patients. Among the studies, fifty-four (54) were cadaveric, while forty-three (43) utilized imaging methodologies and two (2) utilized surgical methods. Concerning the imaging techniques employed, eighteen (18) studies were based on MRA scans, seventeen (17) analyses were carried out using CTA scans, three (3) studies employed DSA, one (1) was carried out using ultrasound scans, one (1) combined CTA, MRA, and DSA scans, and three (3) studies did not report the exact imaging scans used. Regarding the demographics of the studied populations, forty-eight (48) studies were carried out on Asian populations, twenty-four (24) on European populations, twelve (12) on North American populations, six (6) on South American populations, and four (4) on African populations. The characteristics of the included studies are summarized in Table 1.

### 3.3. Morphological Variations in the Anterior Cerebral Artery (ACA)

The typical morphology of the ACA was estimated to have a pooled prevalence of 93.75% (95% CI: 92.20–95.14), while the variant morphology of the ACA was calculated to have a pooled prevalence of 6.25% (95% CI: 4.97–8.00). The distribution of nationality, type of study (cadaveric or radiological), and imaging technique was not statistically associated with the pooled prevalence of the variant morphology (*p* = 0.4857, *p* = 0.1312, and *p* = 0.1291, respectively). The DOI plot indicated an LFK index of +0.94, suggesting no asymmetry and the absence of small-study effects.

The most prevalent variation observed was the aACA, which demonstrated a pooled prevalence of 1.99% (95% CI: 1.50–2.54). The factors of nationality, imaging technique, and patient sex did not significantly influence the estimated prevalence (*p* = 0.6063, *p* = 0.9091, and *p* = 0.2826, respectively). The DOI plot illustrated an LFK index of +0.24 (indicating no asymmetry), suggesting the absence of a small-study effect.

The second most common variation was the absence of the unilateral A1 segment, with a pooled prevalence of 1.78% (95% CI: 1.09–2.62). The nationality distribution, imaging technique, patient’s sex, or side did not influence the pooled prevalence of A1 absence (*p* = 0.2343, *p* = 0.8969, *p* = 0.5992, and *p* = 0.2155, respectively). However, the type of study was a significant factor (*p* < 0.0001), with imaging studies showing a higher pooled prevalence estimate than cadaveric ones (3.59% versus 0.05%, respectively). The DOI plot indicated an LFK index of +0.17 (no asymmetry), suggesting no small-study effect.

The rarest variants included the AzACA, which had a pooled prevalence of 0.22% (95% CI: 0.10–0.36); the fACA was observed at 0.02% (95% CI: 0.00–0.10), and the bACA was also identified at 0.02% (95% CI: 0.02%). No variations were significantly influenced by nationality, imaging technique, side, or the patient’s sex.

### 3.4. Morphometrical Variations in the Anterior Cerebral Artery (ACA)

The pooled mean diameter of the A1 segment was 2.10 mm (95% CI: 1.87–2.34). Furthermore, the hypoplastic A1 segment (with a diameter of less than 1 mm) was found to have a pooled prevalence of 3.15% (95% CI: 2.09–4.40). The nationality, imaging technique, and side factors did not significantly influence the pooled mean diameter (*p* = 0.2226, *p* = 0.2455, and *p* = 0.3098, respectively). The pooled mean length of the A1 segment was approximated at 14.24 mm (95% CI: 12.22–16.25). Insufficient data were available to conduct subgroup analyses for the pooled mean length.

## 4. Discussion

**Figure 2:**
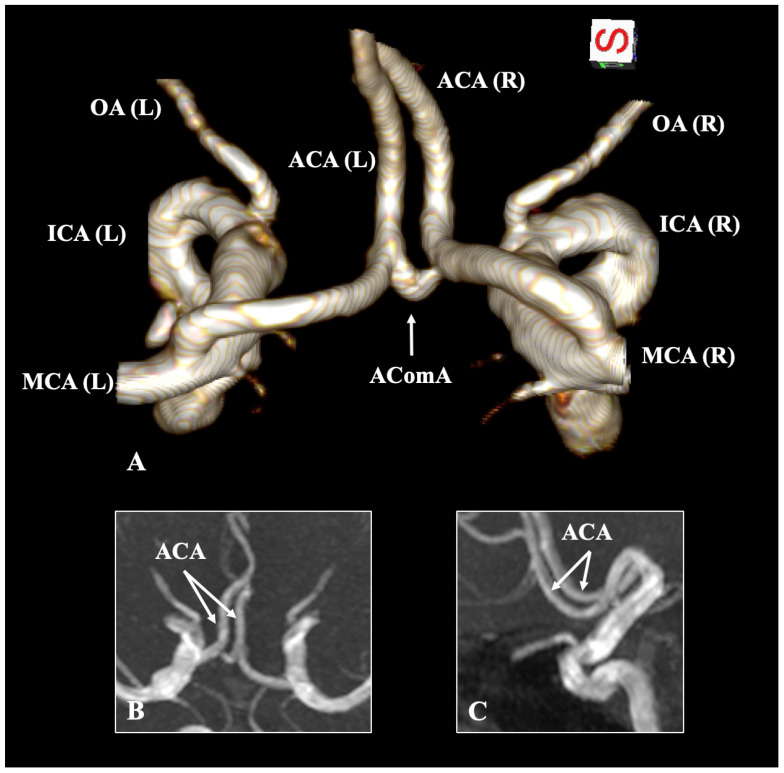
The typical configuration of the anterior cerebral artery (ACA) based on magnetic resonance angiography through three-dimensional (3D) reconstruction (**A**), and in 3D MPR mode coronal (**B**) and sagittal (**C**) slices. ICA—internal carotid artery; MCA—middle cerebral artery; OA—ophthalmic artery; AComA—anterior communicating artery; L—left; R—right.

The present evidence-based meta-analysis examined the variations associated with the ACA, revealing that the atypical configuration occurs in 6.25% of cases, which is considered infrequent, and the typical morphology is 93.75% (Figure 2). Numerous variations exist within the anterior circulation of the brain; however, this review emphasizes the ACA explicitly. The imaging techniques employed did not influence the identification of ACA variants, indicating that MRA, CTA, and DSA are all highly reliable. Other, even rarer variations will be discussed alongside their clinical significance.

**Figure 3:**
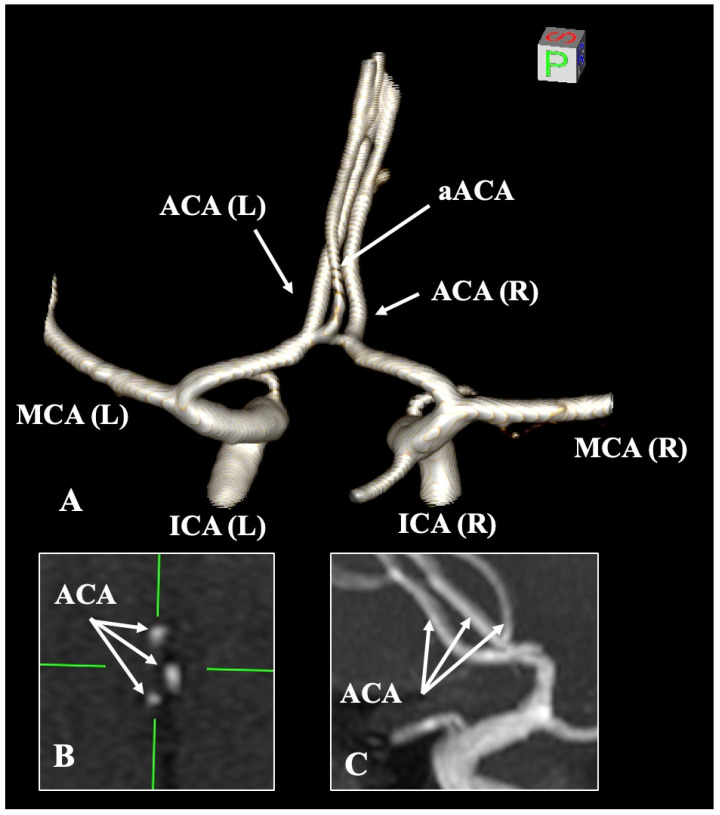
The accessory anterior cerebral artery (aACA) was displayed on magnetic resonance angiography through three-dimensional (3D) reconstruction (**A**), 3D MPR axial slices (**B**), and sagittal slices (**C**). ICA—internal carotid artery; MCA—middle cerebral artery; L—left; R—right.

The aACA is recognized as the most prevalent morphological variant, yielding a pooled prevalence estimate of 1.99% (Figure 3). This variant is commonly referred to by various terms within the current literature, including triplicated ACA, accessory A2 segment, and median artery of the corpus callosum. It is important to highlight that the aACA included in the current meta-analysis had an origin from the AComA, while other origins such as the A1–A2 junction were not included due to the limited data. The imaging modalities employed did not impact the pooled prevalence of this variation; thus, CTA, MRA, and DSA are deemed suitable for diagnosing this variant. Nonetheless, the literature presents varying prevalences attributed to the age demographics of the samples, as older patients with diminished blood flow may possess an aACA that frequently remains undiagnosed [6]. Unfortunately, conducting a subgroup analysis based on age categories for this variant was unfeasible. The clinical significance of this variation pertains to the potential for aneurysm formation at its origin from the AComA [6,7]. In such instances, the aACA is one of the aneurysm’s draining arteries. The trajectory of this variant vessel runs parallel and posterior to the pericallosal artery, rendering it susceptible to intraoperative damage [7]. Notably, Uchino and Tokushige [114] documented the presence of the aACA in conjunction with bilateral supernumerary MCAs. Furthermore, two aACAs (quadriplicated ACA) represent an exceedingly rare variant. Altafulla et al. [115] identified this exceptionally uncommon variation through dissection, where two median arteries of the corpus callosum originated from the AComA.

**Figure 4:**
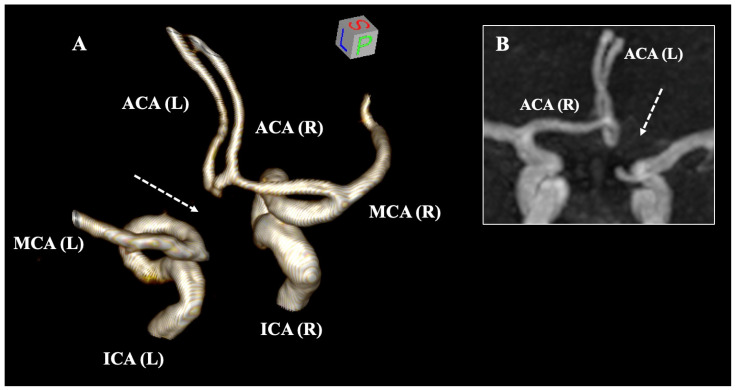
The absent A1 segment (dotted arrows) of the anterior cerebral artery (ACA) is illustrated based on magnetic resonance angiography through three-dimensional (3D) reconstruction (**A**) and 3D MPR coronal slices (**B**). ICA—internal carotid artery, MCA—middle cerebral artery, L—left, and R—right.

The absence of the unilateral A1 segment represents the second most prevalent morphological variant, with a pooled prevalence of 1.78% (Figure 4). A noteworthy detail is that the frequency of this variation is significantly elevated in imaging studies. This can be attributed to the difficulty in distinguishing extreme hypoplasia or potential acquired occlusion on radiological scans, whereas dissection can elucidate even the minutest vessels. Nevertheless, the imaging modality employed (MRA, CTA, or DSA) did not impact the identification of this variant. Two critical clinical implications accompany A1 segment absence: firstly, it induces hemodynamic stress, which frequently leads to the formation of an AComA aneurysm; and secondly, the contralateral A1 segment is often hyperplastic, thereby allowing for easier thrombus entry into this vessel compared to a typical A1 segment [6]. Furthermore, the integrity of the cerebral arterial circle is compromised when one A1 segment is absent; consequently, the primary collateral pathway in instances of stroke is insufficient for establishing collateral circulation [7]. An intriguing case has been documented in which the absence of A1 coincided with bilateral posterior cerebral arteries of fetal origin (originating from the ICA), which entirely disrupted the collateral circulation of the cerebral arterial circle [2].

The unpaired A2 segment is identified as the azACA, a rare variant with a pooled prevalence of 0.22%. In comparison, the bihemispheric A2 segment is recognized when the two segments exhibit asymmetry, characterized by one segment being hyperplastic and supplying the designated territory. In contrast, the hypoplastic contralateral segment exhibits a pooled prevalence of 0.02%. It is essential to acknowledge these two variants distinctly. The presence of an azACA is often associated with the occurrence of distal ACA (dACA) aneurysms [6]. Beyhan et al. [19] categorized the azACA into four distinct types based on its branching pattern. This variant has been previously linked to various conditions, including holoprosencephaly, corpus callosum agenesis, arteriovenous malformation, hydranencephaly, and porencephalic cysts [19]. While our assessment did not establish a significant impact of the imaging technique on the pooled prevalence of the azACA, Beyhan et al. [19] highlighted that CTA should be regarded as the gold standard for diagnosing this variant.

**Figure 5:**
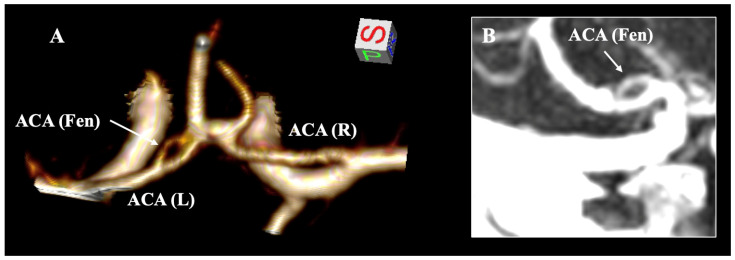
The fenestration (Fen) of the A1 segment of the anterior cerebral artery (ACA) is illustrated in the computed tomography angiography through three-dimensional (3D) reconstruction (**A**) and 3D MPR sagittal slices (**B**). L—left; R—right.

**Figure 6:**
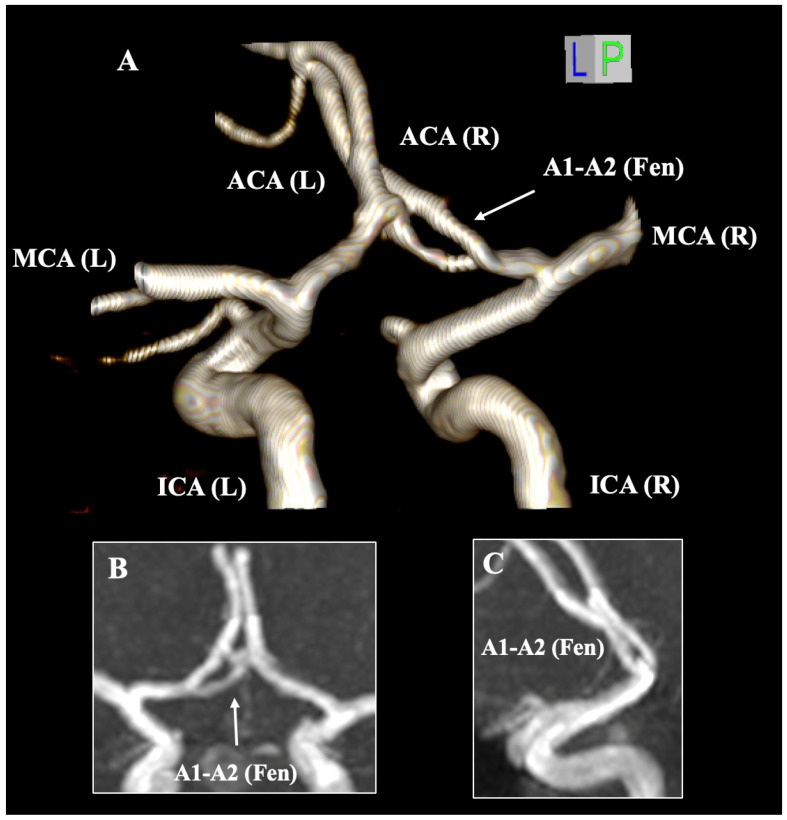
The fenestration (Fen) of the A1–A2 junction of the anterior cerebral artery (ACA) is presented on magnetic resonance angiography through three-dimensional (3D) reconstruction (**A**) and 3D MPR coronal (**B**) and sagittal slices (**C**). ICA—internal carotid artery; MCA—middle cerebral artery; L—left; and R—right.

The fACA was documented to exhibit a pooled prevalence of 0.02% (Figure 5). Within the anterior circulation, the AComA represents the most prevalent fenestration site, with a pooled prevalence of 5% [7]. Nevertheless, the AComA fenestration may be misidentified due to partial or complete duplication and the fenestration at the A1–A2 junction [116] (Figure 6). It must also be distinctly differentiated from the duplicate origin of the ACA, a notably rare variant first described by Uchino et al. [117]. Most studies have reported fenestration at the A1 segment, while fenestration at the A1–A2 junction or the A2 segment is significantly rarer. Uchino et al. [6] identified merely two cases of A2 fenestration in their MRA study, whereas Minca et al. [118] documented one case in their CTA study. Cerebral arterial fenestration is frequently associated with fenestrations at the proximal end, which applies to the ACA. The fenestrated segments exhibit a congenital weakness of the arterial wall, subsequently altering the hemodynamics [6]. Given that the fenestrated branches typically align horizontally, they may be superimposed upon conventional angiographic images; therefore, three-dimensional (3D) imaging data are recommended to identify such variants [6]. Additionally, rarer case reports have indicated instances where fACA is associated with another fenestration within the cerebral arterial circle. Our research team previously described the coexistence of ACA and posterior cerebral artery fenestration [119], as well as the concomitance of ACA and basilar artery fenestration [120].

The morphometric parameters of the A1 segment may possess significant clinical implications, particularly concerning the diameter of the vessel. The pooled mean diameter of the A1 segment is measured at 2.10 mm. When this diameter falls below 1.0 mm, it is classified as a hypoplastic segment. Such variation results in inadequate collateral pathways and is associated with a higher prevalence of ipsilateral hemispheric stroke [22]. Furthermore, the same study noted that A1 hypoplasia constitutes a risk factor for small-artery atherosclerosis [22].

The embryological development of the primitive ICA can elucidate several variants of the ACA. It bifurcates into a cranial and a caudal branch, with the cranial branch representing the future ACA. The cranial branch terminates in the olfactory region and is defined as the primitive olfactory artery (POA). At an embryonic length of 7–12 mm, the precursor to the adult ACA emerges from the POA. At an embryonic length of 12–14 mm, this precursor constitutes the medial branch of the POA, and several plexiform anastomoses exist between the bilateral medial branches, which serve as the precursor to the adult AComA. At an embryonic stage of 20–24 mm, the primitive ACA assumes an upper course between the two cerebral hemispheres. In contrast, several primitive branches have already regressed, and the AComA has not yet reached its definitive form. At this juncture, the AComA exhibits a well-defined superior branch to the corpus callosum, which may persist into adulthood as an aACA (*median artery of the corpus callosum*) [121]. From the embryological development of the primitive ICA, one can observe uncommon and rare types of vessels that persist in the adult cerebral arterial circle. Among them is the persistence of the POA (PPOA), which courses anteriorly along the olfactory nerve and subsequently makes a hairpin turn to continue the typical ACA course. Uchino et al. [122] documented an incidence of 0.14% using MRA, while Kim and Lee [123] identified the PPOA in 0.26% utilizing CTA, and Vasovic et al. [124] reported an incidence of 0.42% through dissections. Notably, intriguing case reports have also emerged, with Radoi et al. [125] describing the coexistence of the PPOA and the azygos pericallosal artery, and Triantafyllou et al. [126] identifying the concomitance of the PPOA, accessory MCA, and early bifurcated ACA. Both cases were documented utilizing CTA. Kim and Lee [123] classified the POA into distinct variants based on its termination and anatomical characteristics: Type 1—the PPOA terminates as a distal ACA; it typically originates from the internal carotid artery, A1 segment, or A1–A2 junction and follows an anterior path along the olfactory tract before turning posteriorly. Type 2—the PPOA ends as the ethmoidal artery, passing through the cribriform plate to supply the nasal cavity. Also, they observed a unique variant of the PPOA that terminates as a distal MCA. This variant is thought to represent remnants of the lateral olfactory branches and was introduced to reflect cases where the PPOA connects to the MCA instead of the ACA. The clinical significance of this rare variation is that aneurysms may develop at the tip of the hairpin turn due to the altered hemodynamics and increased stress at this location [122,125]. Furthermore, its presence should also be anticipated preoperatively for anterior skull base approaches [126]. Another variation that persists from the fetal cerebral arterial circle is the infraoptic course of the ACA, located beneath the ON, which has also been referred to as “carotid-anterior cerebral anastomosis.” Uchino et al. [127] reported an incidence of 0.086% for this scarce variation. Recent studies illustrate that “carotid-anterior cerebral anastomosis” is a scarce variant, while the “infraoptic course” represents a possible morphological and positional variant with a prevalence of 14.48% [128]. “Carotid-anterior cerebral anastomosis” is frequently associated with cerebral aneurysms, particularly at the AComA complex, likely resulting from the hemodynamic stress induced by the abnormal blood flow [127]. Nevertheless, the “infraoptic course” significantly alters the neurosurgical triangles of the skull base, such as the opticocarotid and supracarotid triangles [128]. Therefore, preoperative awareness of this topographical variant of the ACA is of utmost importance, whether through MRA [127] or CTA [128].

Recognizing the limitations of this systematic review is essential, particularly through the lens of meta-analysis. Many included studies demonstrated a notable risk of bias, and several pooled prevalence estimates showed substantial heterogeneity—a common issue across anatomical meta-analyses [9]. Moreover, inconsistent or incomplete reporting of variant laterality and patient sex limited our ability to perform specific subgroup analyses.

## 5. Conclusions

This systematic review and meta-analysis provide an updated, evidence-based synthesis of ACA morphological variations and their pooled prevalence. The typical ACA configuration was observed in 93.75% of cases, with the aACA being the most common variant at 1.99%. While imaging modality influenced prevalence estimates, CTA, MRA, and DSA proved reliable for identifying these variants. Importantly, many ACA alterations are clinically significant due to their association with aneurysm formation and compromised collateral flow. Accurate recognition and documentation of these variants are essential for neuroradiologists and neurosurgeons in diagnostic and preoperative settings.

## Figures and Tables

**Table 1:** The characteristics of the eligible studies, including their risk of bias assessment based on the AQUA tool [13].

Study (Year)	Population	Type of Study	Sample	Patients’ Demographic	Risk of Bias
Ardakani et al. (2008) [16]	Asia	Cadaveric	60	Fetuses and infants (23–74 weeks; mean 48 weeks)	High
Avci et al. (2001) [17]	Asia	Cadaveric	50	Adults	High
Baptista (1963) [18]	South America	Cadaveric	762	NR	High
Beyhan et al. (2020) [19]	Asia	Imaging (CTA/MRA/DSA)	9826	Children and adults (2–86)	High
Bharatha et al. (2008) [20]	North America	Imaging (CTA)	1016	Adults	Low
Chrissikopoulos et al. (2024) [21]	Europe	Imaging (DSA)	912	Adults	High
Chuang et al. (2006) [22]	Asia	Imaging (MRA)	560	Adults	High
Cilliers et al. (2018) [23]	Africa	Cadaveric	78	Adults (22–72)	High
Cui et al. (2015) [24]	Asia	Cadaveric	90	Adults	High
De Silva et al. (2009) [25]	Asia	Cadaveric	450	Adults (18–73)	High
Dharmasaroja et al. (2019) [26]	Asia	Imaging (CTA)	132	Adults	High
Dumitrescu et al. (2022) [27]	Europe	Cadaveric	192	Adults	Low
Dunker and Harris (1976) [28]	North America	Cadaveric	40	Adults (41–83)	High
Eftekhar et al. (2006) [29]	Asia	Cadaveric	204	Adults (15–75)	Low
Fawcett and Blachford (1905) [30]	North America	Cadaveric	1400	Adults	High
Ferre et al. (2013) [31]	Europe	Imaging (CTA)	208	Adults	Low
Fisher (1965) [32]	America	Cadaveric	1428	NR	High
Fredon et al. (2021) [33]	Europe	Imaging (MRA)	1234	Adults (18–65)	High
Furuichi et al. (2018) [34]	Asia	Cadaveric	40	Embryos (end of embryonic period)	Low
Gomes et al. (1986) [35]	North America	Cadaveric	60	Adults	Low
Gunnal (2013) [36]	Asia	Cadaveric	224	NR	High
Halama et al. (2022) [37]	Europe	Imaging (DSA)	556	Adults (17–71)	Low
Hamidi et al. (2013) [38]	Asia	Imaging (CTA)	1000	Children and adults (2–91)	High
Han et al. (2011) [39]	Asia	Imaging (CTA)	334	Adults (mean age 50.9)	High
Hashemi et al. (2013) [40]	Asia	Cadaveric	400	Adults (16–71)	High
Hong et al. (2010) [41]	Asia	Imaging (CTA)	202	Children and adults (13–73)	Low
Huber et al. (1980) [42]	Europe	Imaging (CTA)	15,564	Adults (31–69)	Low
Iqbal et al. (2013) [43]	Asia	Cadaveric	100	NR	High
Jain (1964) [44]	America	Cadaveric	600	NR	High
Jimenez-Sosa et al. (2017) [45]	South America	Imaging (CTA)	566	Children and adults (1–99)	Low
Kahilogullari et al. (2008) [46]	Asia	Cadaveric	60	Adults	High
Kamath (1981) [47]	Asia	Cadaveric	200	NR	High
Kannabathula et al. (2017) [48]	Asia	Cadaveric	150	NR	High
Kapoor et al. (2008) [49]	Asia	Cadaveric	2000	Children and adults	Low
Karatas et al. (2015) [50]	Asia	Cadaveric	200	Adults (16–95)	Low
Kaspera et al. (2014) [51]	North America	Imaging (CTA)	350	Adults (18–75)	Low
Kayembe et al. (1984) [52]	Asia	Cadaveric	88	Adults	High
Kedia et al. (2013) [53]	Asia	Cadaveric	30	Adults	High
Klimek-Piotrowska et al. (2016) [54]	Europe	Cadaveric	200	Adults	Low
Kondori et al. (2017) [55]	Asia	Imaging (MRA)	1050	Adults (25–78)	Low
Kovac et al. (2014) [56]	Europe	Imaging (CTA)	910	Adults	Low
Krabbe-Hartkamp et al. (1998) [57]	Europe	Imaging (MRA)	300	Adults	High
Krystiewicz et al. (2021) [58]	Europe	Cadaveric	666	Adults	Low
Krzyzewski et al. (2015) [59]	Europe	Imaging (CTA)	822	Adults	Low
Kulenovic et al. (2003) [60]	Europe	Cadaveric	200	NR	High
Kwak et al. (1980) [61]	Asia	Imaging (CTA)	592	NR	High
Kwon et al. (2005) [62]	Asia	Imaging (MRA)	482	Adults	Low
Lee et al. (2017) [63]	Asia	Imaging (CTA)	1560	Adults	High
Lehecka et al. (2008) [64]	Europe	Imaging	202	NR	High
LeMay and Gooding (1966) [65]	North America	Cadaveric	214	NR	High
Lopez-Sala et al. (2020) [66]	Europe	Imaging (CTA)	852	Adults	High
Macchi et al. (1996) [67]	Europe	Imaging (MRA)	200	Adults	High
Madkour (2023) [68]	Asia	Imaging (MRA)	148	Adults	High
Malamateniou et al. (2009) [69]	Europe	Imaging (MRA)	188	Neonates (25–35 weeks)	High
Marinkovic et al. (1990) [70]	Europe	Cadaveric	52	Adults	High
Mishra et al. (2004) [71]	Asia	Cadaveric	100	NR	High
Nathal et al. (1992) [72]	Asia	Surgery	268	NR	High
Nordon and Rodrigues (2012) [73]	South America	Cadaveric	100	Adults	Low
Nowinski et al. (2009) [74]	Asia	Imaging (MRA)	96	NR	High
Nyasa et al. (2021) [75]	Africa	Cadaveric	48	Children and adults (3–65)	Low
Ogawa et al. (1990) [76]	Asia	Surgery	412	NR	High
Ogengo et al. (2019) [77]	Africa	Cadaveric	436	Adults	High
Orandogen et al. (2016) [78]	Asia	Imaging (DSA)	256	NR	High
Ozaki et al. (1977) [79]	Asia	Cadaveric	292	All ages (13 h after birth to 88 years old)	High
Papantchev et al. (2013) [80]	Europe	Cadaveric	500	Adults (18–91)	High
Pashaj et al. (2013) [81]	Europe	Imaging (US)	904	Fetuses (18–41 weeks)	High
Perlmutter and Rhoton (1978) [82]	North America	Cadaveric	100	Adults	High
Puchades-Orts et al. (1976) [83]	Europe	Cadaveric	124	NR	High
Qiu et al. (2015) [84]	Asia	Imaging (MRA)	4492	Adults	High
Ring and Waddington (1968) [85]	North America	Cadaveric	50	NR	High
Riveros (2022) [86]	South America	Cadaveric	60	Adults	High
Saha et al. (2024) [87]	Asia	Cadaveric	112	NR	High
Saikia et al. (2020) [88]	Asia	Cadaveric	140	NR	High
Sanders et al. (1943) [89]	North America	Imaging	10,380	Adults	Low
Serisawa et al. (1997) [90]	Asia	Cadaveric	60	Adults	High
Shatri et al. (2019) [91]	Europe	Imaging (MRA)	1026	Adults	Low
Sibiya et al. (2024) [92]	Africa	Imaging (CTA)	478	Adults	Low
Siddiqi (2013) [93]	Asia	Cadaveric	102	Adults	Low
Songsaeng et al. (2010) [94]	North America	Imaging (MRA)	400	Adults	Low
Soundarya et al. (2024) [95]	Asia	Cadaveric	60	Adults	High
Stefani et al. (2000) [96]	South America	Cadaveric	76	NR	High
Stefani et al. (2013) [97]	South America	Imaging (MRA)	60	Adults	Low
Swetha et al. (2012) [98]	Asia	Cadaveric	140	NR	High
Tanaka et al. (2006) [99]	Asia	Imaging (MRA)	234	Adults	Low
Tao et al. (2006) [100]	Asia	Cadaveric	90	Adults	High
Thenmonzhi et al. (2019) [101]	Asia	Cadaveric	200	Adults	High
Tulleken (1978) [102]	Europe	Cadaveric	150	NR	High
Uchino et al. (2006) [6]	Asia	Imaging (MRA)	1782	Children and adults (0–92)	High
Ugur et al. (2005) [103]	Asia	Cadaveric	40	Adults	High
Ugur et al. (2006) [104]	Asia	Cadaveric	100	Adults	High
Van der Zwan et al. (1992) [105]	North America	Cadaveric	50	Children and adults (15–100)	High
Waaijer et al. (2006) [106]	Europe	Imaging (CTA)	206	Adults	High
Wan Yin et al. (2014) [107]	Asia	Imaging (MRA)	7144	Adults	Low
Wijesinghe et al. (2020) [108]	Asia	Cadaveric	146	Adults (51–89)	High
Windle (1888) [109]	Europe	Cadaveric	400	NR	High
Wollschlaeger et al. (1968) [110]	North America	Imaging (DSA)	582	NR	High
Yokus et al. (2021) [111]	Asia	Imaging (MRA)	1162	Adults	Low
Zhao et al. (2009) [112]	Asia	Imaging (MRA)	1524	Children and adults (3–88)	High
Zurada et al. (2010) [113]	Europe	Imaging (CTA)	230	Children and adults (12–78)	Low

## Data Availability

All the data are available upon reasonable request to the corresponding author.

## Supplementary Materials

The following supporting information can be downloaded at: https://www.mdpi.com/article/10.3390/diagnostics15151893/s1, Table S1: PRISMA 2020 Checklist.
